# Abnormal eyeblink conditioning is an early marker of cerebellar dysfunction in preclinical SCA3 mutation carriers

**DOI:** 10.1007/s00221-018-5424-y

**Published:** 2018-11-14

**Authors:** J. van Gaalen, R. P. P. W. M. Maas, E. F. Ippel, M. W. Elting, K. Y. van Spaendonck-Zwarts, S. Vermeer, C. Verschuuren-Bemelmans, D. Timmann, Bart P. van de Warrenburg

**Affiliations:** 1 Department of Neurology, Donders Institute for Brain, Cognition and Behaviour, Radboud University Medical Center, PO Box 9101, 6500 HB Nijmegen, The Netherlands; 2 Department of Medical Genetics, University Medical Center, Utrecht, The Netherlands; 3 Department of Genetics, VU University Medical Center, Amsterdam, The Netherlands; 4 Department of Genetics, Academic Medical Center, Amsterdam, The Netherlands; 5 Family Cancer Clinic, Netherlands Cancer Institute, Amsterdam, The Netherlands; 6 Department of Genetics, University of Groningen, University Medical Center Groningen, Groningen, The Netherlands; 7 Department of Neurology, University Hospital Essen, University of Duisburg-Essen, Essen, Germany

**Keywords:** Eyeblink conditioning, SCA3, Preclinical carriers, Cerebellar dysfunction

## Abstract

**Background:**

Spinocerebellar ataxias (SCAs) are a group of autosomal dominantly inherited degenerative diseases. As the pathological process probably commences years before the first appearance of clinical symptoms, preclinical carriers of a SCA mutation offer the opportunity to study the earliest stages of cerebellar dysfunction and degeneration. Eyeblink classical conditioning (EBCC) is a motor learning paradigm, crucially dependent on the integrity of the olivocerebellar circuit, and has been shown to be able to detect subtle alterations of cerebellar function, which might already be present in preclinical carriers.

**Methods:**

In order to acquire conditioned responses, we performed EBCC, delay paradigm, in 18 preclinical carriers of a SCA3 mutation and 16 healthy, age-matched controls by presenting repeated pairings of an auditory tone with a supraorbital nerve stimulus with a delay interval of 400 ms.

**Results:**

Preclinical carriers acquired significantly less conditioned eyeblink responses than controls and learning rates were significantly reduced. This motor learning defect was, however, not associated with the predicted time to onset.

**Conclusions:**

EBCC is impaired in preclinical carriers of a SCA3 mutation, as a result of impaired motor learning capacities of the cerebellum and is thus suggestive of cerebellar dysfunction. EBCC can be used to detect but probably not monitor preclinical cerebellar dysfunction in genetic ataxias, such as SCA3.

**Electronic supplementary material:**

The online version of this article (10.1007/s00221-018-5424-y) contains supplementary material, which is available to authorized users.

## Introduction

The dominantly inherited spinocerebellar ataxias (SCAs) are progressive neurodegenerative disorders that have cerebellar ataxia as the main clinical theme. Preclinical carriers of a SCA mutation can already display subtle symptoms suggestive of neuronal dysfunction long before the actual manifestation of ataxia (Schols et al. 2004). Such carriers thus enable us to study very early stages of cerebellar degeneration. Indeed, some studies have demonstrated cerebellar dysfunction in preclinical carriers of a SCA mutation (Maas et al. 2015), but this has not led to the availability of a simple tool that is able to detect and track cerebellar dysfunction at the individual level. Such surrogate markers are urgently needed, given the emergence of intervention studies in SCAs.

Eyeblink classical conditioning (EBCC) is a tool to assess cerebellar-dependent associative learning and has been shown to be sensitive to detect subtle cerebellar abnormalities (Bracha et al. 2009; Gerwig et al. 2014).

This is tested for by presenting repeated pairings of a conditioned stimulus (CS; most commonly an auditory or visual stimulus) and an unconditioned stimulus (US; usually an electrical stimulus or air puff to the eye), the latter giving rise to reflexive eyelid closure (unconditioned response, UR). Owing to intact cerebellar motor learning capacity, healthy individuals eventually display a conditioned response (CR; eyelid closure) before onset of the US. The underlying cellular mechanisms of eyeblink conditioning are a matter of current debate. Purkinje cells in the cerebellar cortex show a spontaneous firing pattern. This pattern can be modulated, for instance, by conditioning, which leads to suppression of activity of the Purkinje cells, resulting in a pause of the firing pattern due to learning. As a result, there is less tonic inhibition of the deep cerebellar nuclei that allows for a CR to occur. Recent studies show that the underlying plasticity goes beyond long-term depression (LTD) at the parallel fiber–Purkinje cell synapse. A more complex mechanism involving intrinsic cellular timing also is involved in cerebellar learning (Johansson et al. 2015; Koekkoek et al. 2003; McCormick and Thompson 1984; Schonewille et al. 2011; ten Brinke et al. 2015; Thompson and Steinmetz 2009).

Studies examining EBCC in patients with degenerative ataxias or cerebellar infarction show a significantly decreased incidence and earlier onset of conditioned responses (CRs) (Dimitrova et al. 2008; Gerwig et al. 2003; Perrett et al. 1993; Timmann et al. 2005). It is, however, unknown whether acquisition and timing of CRs are already impaired at the preclinical stage of SCAs. In this study, we characterized EBCC behavior in preclinical carriers of a SCA3 mutation. SCA3 is most common dominant ataxia worldwide and the causative mutation is an expanded CAG repeat in the *ATXN3* gene. Based on the currently available literature, we expected EBCC to be abnormal, but we also wished to evaluate whether this tool could serve as a surrogate marker of disease evolution (Maas et al. 2015).

## Methods

### Subjects

Eighteen preclinical carriers with a proven *ATXN3*/SCA3 mutation and sixteen healthy age-matched controls were included. Carriers were classified as preclinical by an unremarkable neurological examination and a SARA [Scale for the Assessment and Rating of Ataxia, 0 (no ataxia)–40 (severe ataxia)] score of < 3 (Schmitz-Hubsch et al. 2006).

Exclusion criteria were symptoms indicating disease onset such as gait ataxia and/or a SARA score of ≥ 3, a prior history of any neurological disorder, hearing impairment, or use of centrally acting medication. The study was in accordance with the Declaration of Helsinki and carried out with approval of the local ethics committee. All subjects gave written informed consent.

### Eyeblink conditioning

The conditioning stimulus (CS) was an auditory tone (80 dB) presented through binaural headphones, lasting 400 ms. According to a short delay conditioning paradigm, an electrical stimulus with a duration of 200 µs (stimulus width) to the right supraorbital nerve (unconditioned stimulus, US) was given 400 ms after the CS onset.

The US was generated by a Digitimer DS7A (Digitimer Ltd) that stimulated the right supraorbital nerve through a pair of Kendall H59P electrodes (cathode in supraorbital foramen, anode 2 cm above cathode).

The stimulus levels were set at 7–10 times the sensory threshold for the electrical pulse (Blumenthal et al. 2005; Hoffland et al. 2012). The electrical stimuli intensities ranged from 8.0 to 32.0 mA. The paradigm comprised six learning blocks of 11 trials: trial 1–9 were CS–US pairs, trial 10 was US only and trial 11 was CS only. A final, seventh block of 11 CS-only trials was included to assess extinction. Electromyographic (EMG) activity was recorded from both orbicularis oculi muscles.

### Data and statistical analysis

EMG registrations of the orbicularis oculi muscle ipsilateral to the US were analyzed manually. An EMG burst was considered to be a CR in the interval of 150–400 ms after a CS in paired CS–US trials and 150–600 ms after a CS in CS-only trials. In addition, this burst had to last more than 50 ms or be merged into a superimposed blink reflex (unconditioned response, UR, elicited by the US). These bursts were distinguished visually. Peak latency of a CR and UR were measured for the largest amplitude peak of EMG activity with Spike2 software (Cambridge Electronic Design Limited, UK, version 8).

EMG activity within 150 ms after the CS was regarded as an ‘alpha blink’ (startle response) (supplementary data, Figure S1: example of EMG recording).

To analyze CRs, ANOVA was used with BLOCK (Block1, Block2, Block3, Block4, Block5, Block6) as within-subject factor and GROUP (carriers, controls) as between-subject factor. Because of the non-normal distribution of the data (P value Kolmogorov–Smirnov test < 0.05), non-parametric tests (Friedman ANOVA and Mann–Whitney *U* tests) were used to analyze acquisition in and differences between both groups. To assess whether extinction had occurred, the percentages of CRs in blocks 6 and 7 in each group were compared by means of a Chi-square test. In this analysis, only participants that showed at least one CR in the extinction block were included (Gerwig et al. 2006).

To compare means of peak latency of both CR and UR, onset latency of CR, and the number of alpha responses between groups, we used unpaired t tests and Mann–Whitney *U* tests, depending on the distribution of data.

Furthermore, correlations between percentage of CRs in blocks 1–6 and the number of CAG-repeats, SARA score, and time to manifestation (TTM) were assessed. TTM was determined by subtracting current age from predicted age at onset. The latter was calculated using the formula (van de Warrenburg et al. 2005):

$${\text{Lo}}{{\text{g}}_{10}}\left( {{\text{age at onset}}} \right){\text{ }}={\text{ }}3.18{\text{ }}-{\text{ }}0.022{\text{ }}\cdot{\text{ number of CAG-repeats}}.$$

All statistical analyses were conducted using SPSS for Windows (version 22) and significance level was set at 0.05.

## Results

**Fig. 1:**
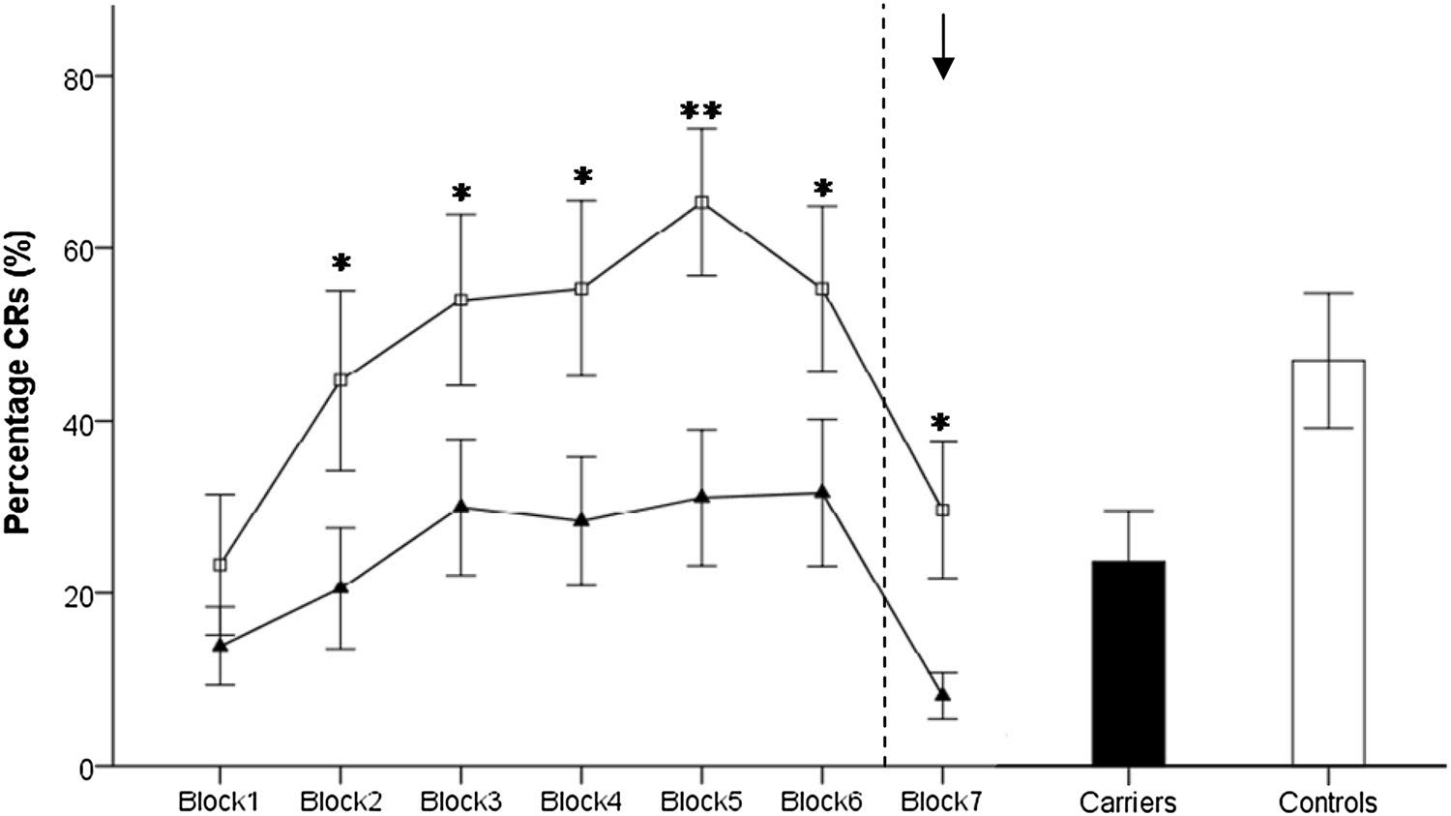
The percentages of conditioned responses in each block of testing (including block 7, the extinction block, indicated by black arrow) are shown for preclinical SCA3 mutation carriers (black triangles) and controls (blank squares). Mean percentage CR incidence over the six acquisition blocks is also shown in a bar diagram on the right to visualize overall performance. Error bars represent standard deviation. *p < 0.05 and **p < 0.005. *CR* conditioned responses

Participant characteristics are summarized in Table 1. Figure 1 displays the acquisition of CRs in both groups. The controls showed a significant learning curve, reflected by an increase of CR percentage during the six learning blocks (*X*^2^ (5) = 43.94, *p* < 0.001), while there was only a trend towards such a block effect for preclinical carriers (*X*^2^ (5) = 10.14, *p* = 0.07). Significant differences between carriers and controls could be demonstrated in total CR percentage over six blocks (*U* = 76.5, *Z* = − 2.33, *p* = 0.02) as well as in mean CR percentage in each block, except for the first.

**Table 1:** Characteristics of preclinical SCA3 mutation carriers and controls

	Carriers (*n* = 18)	Controls (*n* = 16)	*p* value
Age	37.7 years (SD 9.7, range 24–61)	38.5 years (SD 11.1, range 23–58)	0.82
Gender	9M/9F	10M/6F	0.46
SARA score	1.4 (SD 1.0, range 0–2.5)	0.3 (SD 0.4, range 0–1)	< 0.05
Time to manifestation	15.8 years (SD 10.6, range − 1.5 to 31)	NA	
Number of CAG-repeats	66 (SD 2.9, range 62–71)	NA	

*NA* not applicable

Timing of CRs did not differ significantly between both groups (supplementary data). To exclude underlying pathology in the response output circuitry, timing of UR was assessed and shown to be not significantly different (*U* = 140.0, *Z* = − 0.14, *p* = 0.89).

**Fig. 2:**
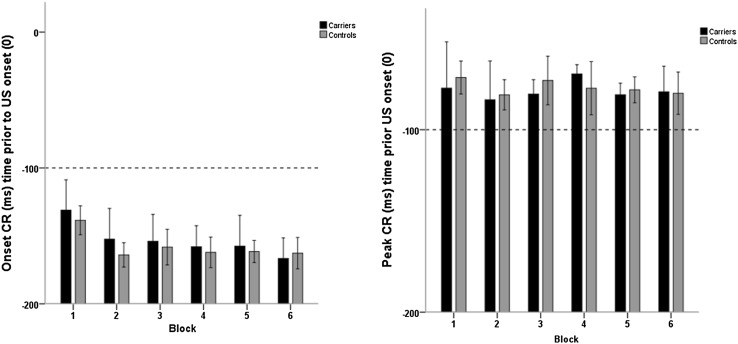
Onset and peak timing of conditioned responses (CR) are shown on the *y* axis. Data are plotted for carriers (black) and controls (grey). Error bars represent standard deviation

Peak timing and onset of CRs are plotted in Fig. 2. No significant difference was found between carriers and controls in these timing aspects of the CRs.

Of all subjects, 7 carriers and 11 controls showed at least one CR in the extinction block. In both groups, significant extinction occurred (*p* = 0.008 and *p* = 0.046, respectively). However, more CRs in the extinction block were seen in controls. Since extinction is likely not complete after one block in controls, it is possible that they are on a higher level of extinction than carriers.

A trend towards significance was found in the number of alpha blinks, with a lower frequency in preclinical carriers (*p* = 0.06). There was no significant increase in alpha blinks over six blocks in both groups.

Mean percentage of CRs in block 1–6 was 26.4% (SD 27.7) in carriers and 52.4% (SD 34.8) in controls. It is known that also amongst healthy controls there are poor performers or non-learners. We therefore subdivided the groups into learners, which were defined as subjects with a total CR percentage above the mean CR incidence minus one SD, and non-learners who performed below the aforementioned criterion (Ernst et al. 2016). Eight out of 18 carriers (44.4%) were non-learners versus 3 out of 16 controls (18.7%), *p* = 0.1.

There was no significant correlation between the number of CRs in blocks 1–6 and SARA score (*r* = − 0.18, *p* = 0.47), CAG-repeat number (*r* = − 0.15, *p* = 0.56), or TTM (*r* = 0.08, *p* = 0.75) for the total group of carriers, nor when subdivided into learners and non-learners (Learners: SARA score (*r* = − 0.42, *p* = 0.3), CAG-repeat number (*r* = 0.01, *p* = 0.98), or TTM (*r* = 0.28, *p* = 0.49); non-learners: SARA score (*r* = − 0.09, *p* = 0.79), CAG-repeat number (*r* = 0.52, *p* = 0.12), or TTM (*r* = − 0.0.09, *p* = 0.79). It is noteworthy that EBCC was abnormal in some carriers that had a predicted TTM of more than 20 years (supplementary data, Figure S2).

## Discussion

Our study shows that the delay paradigm of EBCC is at group level impaired in preclinical carriers of a SCA3 mutation. The group of carriers displayed a lower mean number of CRs and a reduced learning effect was established over the consecutive blocks compared to controls. These results suggest that cerebellar learning is already impaired in the preclinical stages of SCA3, providing an objective measure of cerebellar dysfunction in this phase.

Since SCA3 is a slowly progressive neurodegenerative disease the degenerative process has already started in the preclinical phase of the disease. Histological studies in SCA3 patients show degeneration of the deep cerebellar nuclei. Mainly neuronal loss in the dentate nucleus with relative preservation of the cerebellar cortex is seen, in contrast to other spinocerebellar ataxias (Koeppen et al. 2013; Scherzed et al. 2012). The involvement of these nuclei in the pathology underlying SCA3 is the likely cause of the impaired EBCC in preclinical carriers.

Several animal studies show that intact function of the interposed nuclei is needed for adequate conditioning in EBCC. For instance, very small, localized lesions of the interposed nucleus in rabbits completely abolished CR acquisition (Steinmetz et al. 1992). Furthermore, cooling of the interposed nucleus, temporarily impairing its function, lead to disruption of previously acquired CRs in rabbits. After warming the interposed nucleus back to normal temperature, restoring its function, CRs re-occurred (Clark et al. 1992).

In healthy humans, a 7T functional MRI study showed a concurrent increase in blood oxygenation level-dependent (BOLD) activity of the cerebellar nuclei as well as of the cerebellar cortex in the early phase of acquisition of CRs. Activation was mainly seen in lobules VI of the cerebellar cortex, the interposed nuclei, and also to a lesser extend in the dentate nuclei (Thurling et al. 2015). This suggests an additional contribution of the lateral cerebellum, which projects to the deep cerebellar nuclei, and of the dentate in human EBCC. In SCA3, SWI sequences showed a volume reduction of the dentate nuclei on 7T brain MRI. This is in accordance with the involvement of the dentate in SCA3 as seen in neuropathological studies (Stefanescu et al. 2015). Therefore, our data could suggest that the cerebellar nuclei, in particular the dentate and interposed nuclei, are possibly undergoing early neuronal dysfunction or degeneration in SCA3, as shown by impaired EBCC in preclinical carriers.

Abnormal EBCC has also been demonstrated in patients with symptomatic cerebellar disease. Previous studies have shown that cerebellar degeneration and lesions lead to impaired acquisition of CRs. A patient with primary cerebellar agenesis acquired only a very low percentage of CRs despite 7 days of extensive training (Wu et al. 2018). Patients with cerebellar degeneration showed a prominently reduced number of CRs and a smaller learning effect over consecutive blocks compared to controls. These findings were less severe in patients with focal cerebellar lesions, but acquisition of CRs was still significantly reduced compared to healthy controls (Ernst et al. 2016; Gerwig et al. 2010; Yeo et al. 1985). The preclinical carriers in our study showed similar results with EBCC, although to a less severe extent at the group level. Compared to controls, they acquired a lower number of CRs and cerebellar learning was also limited compared to healthy non-carriers. However, at the individual level, some carriers still displayed normal conditioning, whilst others showed impairment comparable to results found in patients with symptomatic cerebellar disease. This suggests that impairment of EBCC is likely a static trait, in which EBCC is either disturbed or normal in subjects with a certain level of cerebellar dysfunction, instead of a marker of gradual decline.

There was no correlation between the calculated time to manifestation and acquisition of CRs. There might be variability in the point in time when certain sets of neurons, such as those crucial for the EBCC circuitry, start to be dysfunctional in the long preclinical window. Other genetic, environmental, and compensatory mechanisms are important contributing factors to such variability (Falcon et al. 2016). We saw clearly abnormal EBCC even in some carriers with a predicted time to onset of more than 20 years, and conversely normal EBCC in some who were close to their predicted onset. Assuming that EBCC will never improve once abnormal, this also supports our hypothesis that abnormal EBCC would be a static trait, present after exceeding of a certain threshold of dysfunction or degeneration. This is in accordance with another study in patients with cerebellar degeneration that found only weak correlations between clinical ataxia scores and impaired learning with EBCC and the absence of significant worsening over a 1-year follow-up (Timmann et al. 2005).

In contrast to symptomatic patients with degenerative cerebellar ataxia, timing of CRs and extinction were not impaired in preclinical SCA3 mutation carriers (Gerwig et al. 2005). In rabbits, lesions of the cerebellar cortex disturbed the timing of CRs, especially lesions of the anterior lobe (Perrett et al. 1993). Patients with dyslexia with suspected mild cerebellar dysfunction showed, despite normal acquisition and amplitudes of CRs, disturbed timing. This finding suggests possible separate mechanisms for acquisition versus timing of CRs (Nicholson and Freeman 2002). An accurately timed CR is depended on the processes of long-term depression and long-term potentiation, but it is still not clear how this exactly leads to adequate timing (Freeman and Muckler 2003; Racine et al. 1986). Possibly, a temporal relation between the input of the climbing fibers causing depression or potentiation lead to an activity peak in the granule cells, with the same temporal pattern after learning. This then will lead to an adequately timed CR (Johansson et al. 2014). Since we did not find disturbed timing of CRs, but rather a lower number of acquired CRs, acquisition and timing seem to be two distinct processes that can be affected independently.

Extinction was normal in preclinical carriers compared to controls. In patients with focal cerebellar lesions or pure cortical cerebellar degeneration and diminished acquisition of CRs, extinction was found to be impaired (Gerwig et al. 2006). This finding was challenged by a more recent study that showed that in patients with focal cerebellar lesions with sufficient acquisition of CRs (learners) to evaluate extinction, this process was intact, whilst in the majority of patients the number of acquired CRs was too low (non-learners) to draw conclusions about the extinction phase (Ernst et al. 2016). Some studies suggest a cortical process for extinction at the level of Purkinje cells, within an intact cerebellar network (Jirenhed et al. 2007). Since in SCA3 the cerebellar cortex is relatively spared, normal extinction in SCA3 carriers found in our study could favor the hypothesis of an important role of the cerebellar cortex in extinction.

For unconditioned responses (UR), animal studies show contrasting results, but in humans with cortical lesions of the superior cerebellum, increased duration and amplitudes of unconditioned responses were found. In our study, we did not find a difference in UR timing between carriers and controls. This may indicate that this region is not affected in presymptomatic carriers and that cerebellar dysfunction is responsible for the abnormalities in alpha blinks and CRs in carriers, and that there is no other ‘driver’ of these results.

A possible limitation is the inclusion of a relatively small number of subjects; it is therefore difficult to draw definite conclusions about for example extinction. But since SCA3 is a rare disease, and presymptomatic carriers are not very frequently identified, the total number of carriers included in this study is sufficient to study EBCC in this group.

In conclusion, our study provides evidence of abnormal cerebellar motor learning in the preclinical stage of SCA3 mutation carriers and demonstrates that the delay paradigm of EBCC is a relatively simple tool to assess this. However, at the individual level, there is variability in EBCC performance among carriers, irrespective of predicted time to disease onset. Therefore it can be mainly used to assess performance at group level, similar to patients with symptomatic cerebellar disease who also show variability in the level of conditioning. This, combined with the notion that abnormal EBCC is very likely a static trait, questions its utility as a surrogate marker in future treatment trials targeting (preclinical) carriers of a SCA3 mutation.

## Electronic supplementary material

Below is the link to the electronic supplementary material.

Supplementary material 1 (PDF 379 KB)
