# Supplementary material for: Abnormal eyeblink conditioning is an early marker of cerebellar dysfunction in preclinical SCA3 mutation carriers
Source: Exp Brain Res. 2018 Nov 14;237(2):427–33. doi: 10.1007/s00221-018-5424-y (PMC6373441; doi:10.1007/s00221-018-5424-y)

Supplementary data - Results of statistical analyses

Figure S1

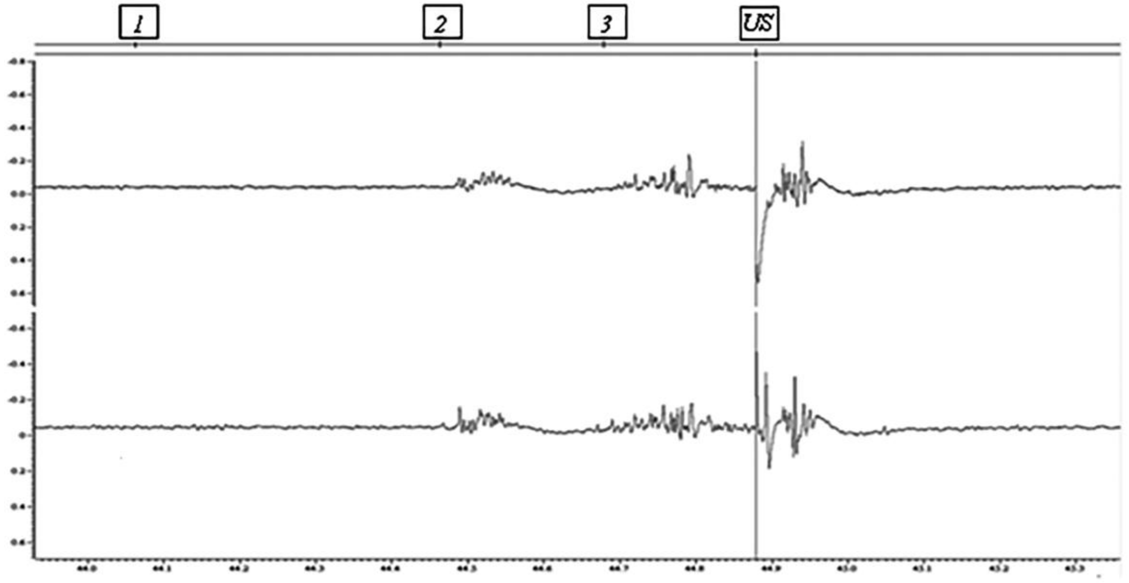

Example of EMG recording.

EMG signal of orbicularis oculi muscle left (upper) and right (lower) of one of the subjects.

1. 400 ms prior to the conditioning stimulus (CS). 2. Time of onset of CS. 3. 200 ms after CS.

Activity in the interval between 200 ms after CS (3) until US is a conditioned response (CR). The activity directly after the US (lamellar artefact) is the unconditioned response (UR). Activity in the interval between 2 and 3 is an alpha-blink (latency < 150 ms and amplitude > 50 µV)

Numbers supporting figure 1

	CRs block 5&6	CRs Block 1-6	Block1	Block2	Block3	Block4	Block5	Block6	Block7
Mann-Whitney U	72,500	76,500	124,000	84,000	81,500	73,000	60,000	79,500	72,000
Wilcoxon W	243,500	247,500	295,000	255,000	252,500	244,000	231,000	250,500	243,000
Z	-2,481	-2,330	-,739	-2,123	-2,178	-2,478	-2,926	-2,254	-2,372
Asymp. Sig. (2-tailed)	,013	,020	,460	,034	,029	,013	,003	,024	,018
Exact Sig. [2*(1-tailed Sig.)]	,012	,018	,506	,039	,030	,014	,003	,025	,022

Learning effect over blocks: Significant for controls not for carriers

Carriers	N	18
	Chi-Square	10,141
	df	5
	Asymp. Sig.	,071
Controls	N	16
	Chi-Square	43,938
	df	5
	Asymp. Sig.	,000

Post-hoc tests

Group		Block2 - Block1	Block3 - Block2	Block4 - Block3	Block5 - Block4	Block6 - Block5
Carriers	Z	-1,394	-2,104	-,499	-1,020	-,141
	Asymp. Sig. (2-tailed)	,163	,035	,618	,308	,888
Controls	Z	-2,944	-2,310	-,525	-1,983	-2,226
	Asymp. Sig. (2-tailed)	,003	,021	,599	,047	,026

Timing plus magnitude conditioned responses (CRs): amplitude CRs lower for carriers

	CRs Time to onset Paired	CRs Time to onset CS only trials	CRs Time to peak Paired	CRs Time to peak CS only trials	CRs Amplitude Paired	CRs Amplitude CS only trials
Mann-Whitney U	89,000	70,000	100,000	68,000	59,000	56,000
Wilcoxon W	225,000	175,000	236,000	173,000	179,000	147,000
Z	-1,225	-1,019	-,499	-1,116	-2,411	-1,698
Asymp. Sig. (2-tailed)	,220	,308	,618	,264	,016	,089
Exact Sig. [2*(1-tailed Sig.)]	,232	,325	,637	,280	,015	,094

Timing plus magnitude unconditioned response (UR): no significant difference

	Number of Alpha blinks	URs Time to peak	URs Amplitude
Mann-Whitney U	87,500	140,000	139,500
Wilcoxon W	258,500	311,000	310,500
Z	-1,953	-,138	-,155
Asymp. Sig. (2-tailed)	,051	,890	,877
Exact Sig. [2*(1-tailed Sig.)]	,050	,905	,878

Numbers supporting figure 2

	Onset CR Block 1	Onset CR Block 2	Onset CR Block 2	Onset CR Block 4	Onset CR Block 5	Onset CR Block 6
Mann-Whitney U	25,500	47,000	81,500	74,000	87,500	77,000
Wilcoxon W	61,500	138,000	186,500	194,000	223,500	213,000
Z	-,683	-,362	-,129	-,441	-,395	-,884
Asymp. Sig. (2-tailed)	,495	,717	,897	,659	,693	,377
Exact Sig. [2*(1-tailed Sig.)]	,505	,750	,899	,683	,698	,397
	Peak CR Block 1	Peak CR Block 2	Peak CR Block 3	Peak CR Block 4	Peak CR Block 5	Peak CR Block 6
Mann-Whitney U	25,000	51,000	54,000	78,000	93,500	57,500
Wilcoxon W	61,000	142,000	132,000	144,000	171,500	193,500
Z	-,736	-,072	-1,544	-,234	-,116	-1,788
Asymp. Sig. (2-tailed)	,462	,942	,123	,815	,908	,074
Exact Sig. [2*(1-tailed Sig.)]	,505	,972	,131	,838	,909	,074

Extinction carriers and controls

		Value	df	Asymp. Sig. (2-sided)
Carriers	Pearson Chi-Square	36,000	30	,208
	Likelihood Ratio	27,413	30	,602
	Linear-by-Linear Association	1,833	1	,176
	N of Valid Cases	9		
Controls	Pearson Chi-Square	43,000	36	,196
	Likelihood Ratio	31,407	36	,687
	Linear-by-Linear Association	2,288	1	,130
	N of Valid Cases	12		

Alpha blinks – No significant learning effect over blocks

		alpha2 - alpha1	alpha3 - alpha2	alpha4 - alpha3	alpha5 - alpha4	alpha6 - alpha5
Carriers	Z	-2,377	-1,291	-,280	-1,144	-,187
	Asymp. Sig. (2-tailed)	,017	,197	,780	,253	,852
Controls	Z	-,869	-1,027	-1,184	-,259	-1,125
	Asymp. Sig. (2-tailed)	,385	,304	,237	,796	,261

Figure S2 - Total percentage of CRs acquired in block 1 to 6 related to predicted time to manifestation (TTM, in years).

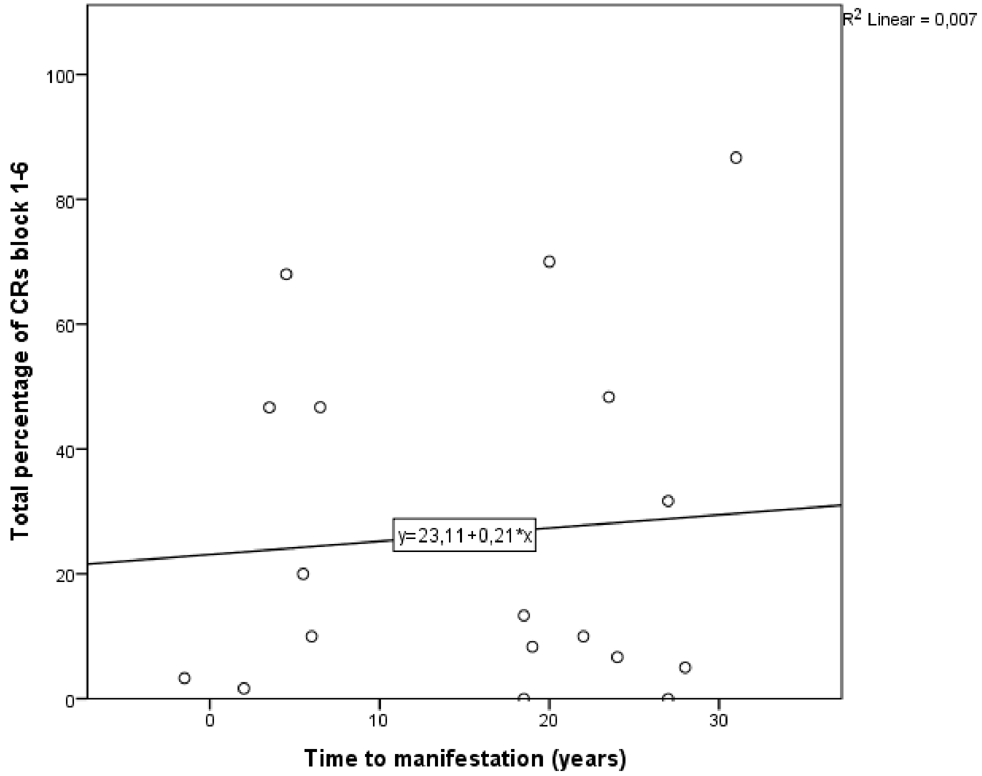

Supplement: Supplementary file 1 — Supplementary material 1 (PDF 379 KB) [file 221_2018_5424_MOESM1_ESM.pdf]
